# Medical Formulæ, with Remarks

**Published:** 1867-02

**Authors:** D. W. Hammond

**Affiliations:** Macon, Ga.


### ARTICLE IV.

*Medical Formulæ, with Remarks.* By D. W. Hammond, M. D., Macon, Ga.

Over the door of one of the most popular restaurants in the city of New York, we find inscribed upon a beautiful sign board the following:

"*Nunquam non Paratus*,"

Which sentiment has been adopted by one of our city druggists, and elegantly engraved on glass in front of the prescription desk. This is a very appropriate motto for every calling and profession. The practising physician, more particularly than all others, as he should *never be unprepared*, should be ready at all times, and in every emergency to conform to it. His business is to grapple day by day with disease and death. Every malady to which the animal organism is subject, is the object of the healing art, the purpose of which is their prevention, cure, or alleviation. The agents which are used by the practitioner are almost entirely pharmaceutic. He should therefore be well versed in medical science and chemical knowledge; for without a thorough acquaintance with the modus operandi of remedial agents and chemical affinities, his formulæ would be, in many instances, incomplete, and instead of relieving his patient of the disease for which he prescribed, would complicate and aggravate it. From this view of the subject, I think every physician should have a note book, in which he should record every important case and the treatment. His prescriptions, when found beneficial, as well as those of his confreres, should be plainly and legibly written.

By pursuing this plan a few years, he will have collected a great number of valuable recipes, which he can readily turn to, and select one to suit many of the cases upon which he may be called on to prescribe. A physician who has a large and extensive practice is necessarily compelled to make "extemporaneous prescriptions," and in the hurry of the moment mistakes sometimes occur. But when he has a variety of well selected prescriptions, prepared by himself or others, recorded in a book kept for the purpose, if one is found suited to the case upon which he is consulted, it can be copied without any danger of committingan error. It is common in France, and the course pursued by Griffith, to write prescriptions in *words* instead of the usual pharmaceutic signs. I am of opinion, however, that mistakes are as apt to take place when the prescription is written, as when expressed by signs.

I, therefore, write all my prescriptions by the old abbreviated cabalistick terms.

To demonstrate the practical utility of keeping a recipe book, I will now give a case or two.

A patient presents himself for medical advice, stating that his "stomach is out of order;" complains of heartburn, acid eructations, vomiting occasionally after eating, and frequent paroxysms of sick headache, mouth slimy and rather dry, some pain in the small of the back, urine scant and highly colored, bowels costive, etc. Turn to your note book, under the head of Dyspepsia, where you know you have the remedy to suit his case. Copy recipe No. 1, and direct him how it is to be taken. Another calls to consult you, stating that he has been subject to frequent recurring intermittents for nearly twelve months. He will tell you that his health, notwithstanding, is tolerable good, and that the chills are slight and short in duration, with slight fever and some headache, and pain in the loins after each paroxism.

On examination, you will find his tongue foul, and his eyes slightly jaundiced.

This form of chronic chills is caused by a neuropathic condition of the system, and more properly denominated *nervous rigors.* The appropriate prescription in such case you will find under the head of "Chronic Chills," Nos. 2 and 3, and also the directions how to administer them.

DYSPEPSIA.

No. 1. ℞ Pv. Rhei,

Sup. Carb. Soda, ā ā ʒ ii.

Carb. Potass ....... ℈ ii.

Aqua Camphora.... ℥ viii.—Mix.

Dose, a tablespoonful 15 minutes before eating.

If I were restricted to a single remedy in the treatment of this disease, I would prefer this to any other I have used, and for the following reasons: The soda, and also the potassa are both antacids. The former is eliminated through the salivary glands, and the latter through the kidneys. When the saliva is deficient, the gastric juice becomes acrid. The soda increases the salivary secretion, thereby dilutes it. In this way the acid is neutralized, and the irritation of the mucous membrane of the stomach is relieved. The kidneys being the emunctory through which the potassa is eliminated, are restored to their normal condition. The rhubarb is a subtonic and laxative, and prevents costiveness. The camphor water relieves gastrodinia, and expells the flatus which is always in excess in dyspepsia. To be taken without shaking the vial.

CHRONIC CHILLS (OR FREQUENT RECURRING INTERMITTENTS).

No. 2 ℞ Infusum Eupatorii Perfoliati........ ℥ vi.

Tr. Valerian..................... ℥ ii.

Tr. Nux Vomicie................... ʒ ii

Sulph. Quinine.................... ℈ ii

Acid Sulph. Aromat. Q. S. —M.

A tablespoonful three times a day. On the day of chill, three or four doses should be taken an hour apart, and the last dose one hour before the chill is expected to come on. When the patient is feeble and anemic, and troubled with neuralgic throbbings in the temples, Fowler's solution of arsenic (℥ i) shsuld be added to the formula, and taken after meals.

No. 3. ℞ Comp. ext. Colocynth,
Blue Mass............ ā ā grs. 15
Sulph Quinine............grs. 25

Mix and make 24 pills. Two to be taken every two hours, the day after the chill. When the patient has a yellowness of the skin and eyes, with hepatic derangement, this is an invaluable remedy.

No. 4. ℞ Acid Arseniosi................grs. iss.

Quinine Sulph.

Ferri. Sulph. ā ā ............ ʒ i.

Sulph. Morphine..............grs. i.

Ext. Nux Vomica............... ℈ i. Gross.

M. ft. 30 pills.

Sig.—One to be taken every five or six hours. A good remedy when the patient is anemic, and troubled with neuralgic headache.

No. 5. ℞ Comp. Tr. Gentian............. ℥ iij.
Tr. Columbo
Comp. Tr. Cardaman .........ā ā ℥ ss.
Strychnine ............grs. i.
Sulph. Morphine....... " ii.—Mix.

A teaspoonful at bed-time, and 2 next day, preceding the chill. I have found the above to answer well in persons who could not take quinine.

COSTIVENESS.

No. 6. Ext. Hyoscyamus.................ʒi.

Comp. Ext. Colocynth.............℈ij.

Ext. Nux Vomica.............grs. vi.

M. ft. Pill No. 24.

One taken every night generally gives a loose operation next morning. These pills are very mild in their action. The nux vomica appears to heighten the action of other cathartics, while the hyoscyamus prevents griping.

No. 7. ℞ Ext. Belladonna....grs. 2
Ext. Glycyrrhiza......qs.—M. ft. Pills No. 8.

Prof. Trousseau says when you wish merely to assist defication, without purging, that "belladonna is the remedy *par excellance.*"

No. 8. ℞ Extract Belladonna...gr. i.
Pv. Rhei..........gr. 15
Pv. Ipecac........gr. 5—M. ft. Pills No. 5.

These pills have long been known as Dr. Chapman's Peristaltic Persuaders. I have found them an excellent pill. They are frequently taken after eating a hearty dinner, by epicures. One is a dose.

No. 9. ℞ Ext. Nux Vomica,

Ext. Belladonna,............āā grs. x.

Comp. Ext. Colocynth............ʒij.

Aloes. Socat.......................℈i.

M. and make 40 pills.

These are mild in their action and more active than No. 8. One night and morning when the bowels are torpid.

No. 10. ℞ Strychnine..............gr. ss.

Ext. Colocyth Compos..... ʒss.

M. ft. pills 30.

One to be taken, 3 times a day. They increase virmicular action.

No. 11. ℞ Mag. Sulphas............... ℥ 8.

Elixir Vit............... f. ℥ i.

Tr. Aurantii. ............. f. ℥ 6.

Atropine....................gr. i.

Aqua Font.................. ℥ 53.—M.

Dose, ℥ i. on going to bed.

When the bowels are sluggish, and the inner lining loaded with mucous, there is no superior remedy to this. It should not be continued too long at any one time, as it dissolves the mucous coating and acts as an irritant.

DIARRHŒA.

No. 12. ℞ Pv. Rhei.,

Cal. Magnesia, .............āā ʒ ss.

Syrup Red Pepper............ ℥ iss.

Sulph. Morphine.............grs. ii.

Aqua Menthæ................ ℥ vi.

Pv. Gum Arabic.............. ʒ iij.—Mix.

A desertspoonful 3 or 4 times a day. Shake the vial before using. This is pretty much the same as Waggamans' Mixt., and suited for billious diarrhœa, or when it is kept up by substances offending the stomach and bowels.

No. 13. ℞ Spts. Turpentine............. ʒ iss.

Pv. G. Arabic.............. ℥ ss.

Carb. Magnesia..............℈ ij.

Tr. Opii.................... ʒ i.

Aqua Camphora.............. ℥ vi.—Mix.

A tablespoonful 3 or 4 times daily. Diarrhœa supervening in typhoid fevers, with tympanitic dystention, and hyperæsthesis of the mucous membrane of the large bowels. Turpentine and opium should never be omitted.

ANOTHER.

No. 14. ℞ Sub. Nit. Bismuth............ ℥ i.

Tr. Catachu................... ℥ i.

Tr. Opii. ..................... ℥ iss.

Syrup Ginger................. ℥ v.—Mix.

Sig.—A tablespoonful pro re nater.

No. 15. ℞ Tr. Opii,

Spts. Camphor,

Tr. Capsicum,

Tr. Myrrh,.................. ā ā ℥ i.

Tr. Catachu .................... ℥ ij.—M.

A teaspoonful, according to the urgency of the case, in a wineglass of water. Used in chronic cases. It has been extensively used in the army. Dr. Dugas, of Augusta, is the author of it.

No. 16. ℞ Oxide Zinc.......... ʒ i.

Crumb of Bread..... qs.—M. ft. pills 20.

One to be given morning, noon and night. I, on one occasion, prescribed this preparation of zinc in a case of epilepsy, complicated with an intractable diarrhœa. The epileptic convulsions and diarrhœa were both cured, which I attributed to the arrestation of the bowel affection.

No. 27. ℞ Acid Tannic.................. ʒ v.

Glycerin...................... ℥ iv.

Creosote ...................gtts. xvi.

Ol. Sassafras ...............gtts. v.—M.

A teaspoonful for an adult. In gastric and intestinal hemorrhage, resulting from hæpatic obstructions, where the dejections are black like tar, I have prescribed this formulæ repeatedly, with success.

ERYSIPELAS.

No. 18. ℞ Sulph. Magnesia......... ℥ iij.

Aqua Font.............. ℥ viij.—Mix.

This is the best external remedy I have ever used. I commenced the use of it in the year 1845. It should be applied to the part three or four times a day, and suffered to dry on the skin, which leaves a white powder: this serves as a coating. Best to apply as warm as the patient can bear it, as the vitality of the part affected with erysipelas is always lowered. I am well aware that many contend that erysipelas is a symptomatic disease, and not local, or idiopathic. Hence, it being unsettled, and still a subject "*sub lite*," it is not my province here to consider its ætiology. But one thing I will assert, that a saturated solution of Epsom salts applied *locally* to a part affected with this disease, has arrested it repeatedly, in my hands, without any constitutional medication. And yet, there is another circumstance connected with this disease which I do not understand; and that is, persons affected with it can tolerate four times as much of the muriated Tr. iron on the stomach, as in any other disease. I have frequently given from 40 to 50 drops every 3 or 4 hours, with impunity; and where this is the case, the patient generally recovers.

### DELIRIUM TREMENS.

No. 19. ℞ Tr. Opii.................... ℥ i.

Tart. Antimonii.........grs. ii.

A teaspoonful to be repeated every hour until sleep is induced. Dr. S. Henry Dickson, in a lecture delivered in the Medical College of the State of South Carolina, in order to impress the class with the importance of large doses of opium early in this disease, used the following quotation: "Throw the stone and the giant dies."

I have found exceptions to this rule. We frequently meet with cases where large and repeated doses fail to relieve the insomnia. In persons of this sort, if the remedy is pushed too far, fatal congestion of the brain will inevitably ensue. To prevent which, it will be found a safer treatment to combine tartar emetic with the opium.

No. 20. ℞ Bromide Potassium.....grs. 640
Aqua Menthæ............. ℥ ij.
Aqua Font................ ℥ vi.—Mix.

A tablespoonful every two hours. I have used this prescription in two cases recently. In one case where the opium entirely failed, two doses of 40 grains each of the bromide potassium put the patient to sleep, which continued uninterruptedly for nine consecutive hours. In the other, several doses were required, but in both the disease was rebuked.

No. 21. ℞ Acetate Morphine........grs. x.

Aqua ................... ℥ ss.—Mix.

Twelve drops of the mixture to be injected under the skin, to be repeated as often as may be required. Medicines act much more promptly and efficiently when used hypodermically, than when taken into the stomach. The stomach of the debauchee frequently becomes so irritated that everything taken into it is immediately ejected. Where this is the case, medicine should be given hypodermically.

No. 22. ℞ Pv. Squills...................grs. 60

Pv. Gum Myrrh,

Rubigo Ferri,.................ā ā 80

Hydrarg. Chlorid Mite........grs. 20

Mix ft. pills 30 or 40.

Dose from 5 to 8 daily. A good remedy in anemic condition of the system, with hepatic obstructions. Or in dropsies which are occasioned by dfective absorption. These are passive dropsies, and the right side of the heart is always seriously involved. The pills are powerfully diuretic and alterative, and generally bring on ptyalism. I have known the most extensive anasarca relieved in a few days by these pills. If ptyalism ensues, continue the remedy, omitting the calomel. In giving the pills, I generally direct the patient to drink freely of a decoction of the Calcanthus Floridus, which is also a very powerful diuretic. Most cases of passive dropsy will yield to the above treatment in a short time.

### NEURALGIA.

No. 23. ℞ Hypophosphite Soda......... ℥ i.

Aqua Meuthæ................ ℥ iv.—Mix.

A tablespoonful every three hours.

I was called, a few months since to see a professional brother, who was suffering from a severe attack of facial neuralgia. The uppermost branch of the trifacial nerve appeared to be the seat of the disease. He complained of violent pain in the right eye shooting up into the side of the head. The eye was quite red, closed, and tears streaming from it, photophobia, etc. He said to me that he had "taken everything"—opium, warm applications, chloroform, large doses quinine, and had used leeches freely, and without any abatement of pain. I prescribed hypophosphite of soda, in drachm doses, as above. After three doses had been taken, the pain gradually subsided, and he fell into a quiet slumber, which lasted for several hours. When he awoke the pain was gone, and had no return of it. As is usual in such cases, the drug last given is held to have cured the disease. *Post hoc ergo, propter hoc.*

No. 24. ℞ Tr. Aconite (Flemming's). . . . ℥ ss.

Tr. Opii,

Turpentine Soap,

Chloroform, . . . . . . . . . . . . . . āā ℥ i.

M. ft. linament.

Or,

No. 25. ℞ Tr. Aconite,

Chloroform . . . . . . . . . . . . . . . .āā ℥ i.—M.

These formulæ are applied frequently along the course of the nerve. And afford temporary relief in many instances.

No. 26. ℞ Chinoidine . . . . . . . . . . . . . . . . .24 grs.
Pv. Capsicum . . . . . . . . . . . . . 5 "
Strychnine . . . . . . . . . . . . . . . 1 "
M. ft. pills 12.

Dose—a pill before each regular meal. After the above has been used sufficiently long to break down the paroxisms, the following may be substituted to complete the cure and restore the strength:

No. 27. ℞ Quevennes Iron,
Quinine, . . . . . . . . . . . . . . āā 60 grs.
Ext. Hyoscyamus . . . . . . . . .40 "
Pv. Capsici . . . . . . . . . . . . . . .20 "
Mix and divide into 40 pills.

One pill to be taken after each meal. The above treatment was first recommended by Dr. Robinson, Charleston, So. Ca.

No. 28. ℞ Ext. Conium,

Ext. Hyoscyamus,.......... ā ā ʒ ss.

Sulph. Morphine............grs. ii.

M. Divide into 20 pills.

Sig.—One three times a day.

No. 29. ℞ Cherry Laurel Water.......f. ℥ iv.
Sulph. Ether................ ℥ j.
Ext. Belladonna............. ʒ ij.—Mix.
Rub on the neuralgic site frequently. Mostly used in traumatic cases, in conjunction with the pills, (No. 28.)

No. 30. ℥ Fluid Ext. Canabis Indica.... ʒ i.
Aqua Meuthæ................ ℥ i.—Mix.
A teaspoonful three times a day. I have found it a good remedy where the pain is confined to the head.

No. 31. ℞ Atropine..................grs. i.

Water..................... ʒ vi.—Mix.

No. 32. ℞ Atropine..................grs. i.

Acetate Morphine.........grs. 10

Aqua Font................. ʒ vi.—Mix.

No. 33. ℞ Acetate Morphine.........grs. ii.
Aqua Font.................. ʒ i.—M.
These recipes are to be used hypodermically. I generally inject 12 drops of either of the three. I prefer the external side of the arm, about two inches above the insertion of the deltoid muscle. When the pain is superficial the neuralgic site should be selected for the puncture.

SPERMATORRHŒA.

No. 34. ℞ Strychnine.................grs. i.

Dilut. Nit. Acid............ ℥ iv.—Mix.

A teaspoonful three times a day. It imparts tone to the seminal vessels, and relieves general debility. It should be given in small doses, to prevent its influence on the excito-motory system.

No. 35. ℞ Bromide Potassium.......... ℥ ij.
Comp. Tr. Gentian,
Aqua Eont,..............ā ā ℥ iv.

A desert spoonful three times a day. Spermatorrhœa is often the result of masterbation, and this practice is very often resorted to for the relief of excessive venerial desire (satyriasis). The bromides act as special sedatives upon the reproductive organs. I have been in the habit of giving bromide of ammonia, recently in Gonnorrhœa, in conjunction with other appropriate treatment. Patients who take it are almost entirely exempt from cordie.

No. 36. ℞ Pv. Digitalis..............grs. 20

Divide into twenty powders. One three times daily. Highly recommended by Dr. Laroche.

No. 37. ℞ Tr. Ferri Muriat.,

Tr. Secal. Cornut, ........ ā ā ℥ i.—Mix.

Sig.—Thirty drops three times a day. I have found the above to act well in some cases.

No. 38. ℞ Pv. Nucis Vomicæ............ ℈ ij.

*Vallet's* Proto-Carbonate Iron.. ʒ iij.

Ext. Gentian.................. ʒ iss.

M. ft. pills No. 60. One three times daily.

No. 39. ℞ Emplas Plumbi.............. ℥ ss.

Strychnine................ ℈ ss.

Pices Bergund.............. ʒ ij.

M. ft. Plaster.

To be spread on sheep skin from three to five inches square, and applied to the lower part of the spine. I have used these remedies repeatedly, and with entire success.

WHOOPING COUGH.

No. 40. ℞ Acid Prussic, U. S. D.......guttæ vi.

Ext. Belladonna...............gr. ii.

Tr. Opii Camphorata.......... ʒ iij.

Syrup Tolu.................... ℥ i.

Aqua Font..................... ℥ iij.—Mix.

A teaspoonful every three hours to a child two years old. After removing the complications, (if any,) the above is a favorite remedy with me. It acts well when the disease is somewhat advanced, and spasmodic.

No. 41. ℞ Bromide Ammonium........ ℥ i.

Glycerine,

Syrup Tolu............... āā ℥ iij.—Mix.

Dose to infant from three to six months old, 10 to 20 drops four times a day; from four to six years, a teaspoonful; six to ten, from two to three teaspeonfuls. I consider this formula from the trial I have made of it, an invaluable remedy in whooping cough. I use it early in the disease. It keeps down inflammatory symptoms, and disposes to sleep. The cough is lessened in frequency and in violence. I am of opinion that the remedy abridges the disease at least one half. If expectoration is slight, I generally use the same quantity of Mel. Scillæ Comp., in place of the syrup tolu.

