# Corrections

**Published:** 1867-03

**Authors:** 


### CORRECTIONS.

In the article "Medical Formulæ," by Dr. D. W. Hammond, published in the February number of this Journal, we, by request, make the following corrections: In recipe No. 2, for "recurring intermittents," the quantity of Fowler's solution of arsenic is one drachm, instead of one ounce. In recipe No. 22, "calcanthus" should be "calycanthus."

